# Major Improvements in the Quality of Malaria Case-Management under the “Test and Treat” Policy in Kenya

**DOI:** 10.1371/journal.pone.0092782

**Published:** 2014-03-24

**Authors:** Dejan Zurovac, Sophie Githinji, Dorothy Memusi, Samuel Kigen, Beatrice Machini, Alex Muturi, Gabriel Otieno, Robert W. Snow, Andrew Nyandigisi

**Affiliations:** 1 Malaria Public Health Department, Kenya Medical Research Institute-Wellcome Trust-University of Oxford Research Programme, Nairobi, Kenya; 2 Centre for Tropical Medicine, Nuffield Department of Clinical Medicine, University of Oxford, Oxford, United Kingdom; 3 Center for Global Health and Development, Boston University School of Public Health, Boston, Massachusetts, United States of America; 4 Division of Malaria Control, Ministry of Health, Nairobi, Kenya; 5 Management Sciences for Health, Nairobi, Kenya; Centro de Pesquisa Rene Rachou/Fundação Oswaldo Cruz (Fiocruz-Minas), Brazil

## Abstract

**Background:**

Monitoring implementation of the “test and treat” case-management policy for malaria is an important component of all malaria control programmes in Africa. Unfortunately, routine information systems are commonly deficient to provide necessary information. Using health facility surveys we monitored health systems readiness and malaria case-management practices prior to and following implementation of the 2010 “test and treat” policy in Kenya.

**Methods/Findings:**

Between 2010 and 2013 six national, cross-sectional, health facility surveys were undertaken. The number of facilities assessed ranged between 172 and 176, health workers interviewed between 216 and 237 and outpatient consultations for febrile patients evaluated between 1,208 and 2,408 across six surveys. Comparing baseline and the last survey results, all readiness indicators showed significant (p<0.005) improvements: availability of parasitological diagnosis (55.2% to 90.7%); RDT availability (7.5% to 69.8%); total artemether-lumefantrine (AL) stock-out (27.2% to 7.0%); stock-out of one or more AL packs (59.5% to 21.6%); training coverage (0 to 50.2%); guidelines access (0 to 58.1%) and supervision (17.9% to 30.8%). Testing increased by 34.0% (23.9% to 57.9%; p<0.001) while testing and treatment according to test result increased by 34.2% (15.7% to 49.9%; p<0.001). Treatment adherence for test positive patients improved from 83.3% to 90.3% (p = 0.138) and for test negative patients from 47.9% to 83.4% (p<0.001). Significant testing and treatment improvements were observed in children and adults. There was no difference in practices with respect to the type and result of malaria test (RDT vs microscopy). Of eight dosing, dispensing and counseling tasks, improvements were observed for four tasks. Overall AL use for febrile patients decreased from 63.5% to 35.6% (p<0.001).

**Conclusions:**

Major improvements in the implementation of “test and treat” policy were observed in Kenya. Some gaps towards universal targets still remained. Other countries facing similar needs and challenges may consider health facility surveys to monitor malaria case-management.

## Introduction

The paradigm shift from presumptive treatment of fevers to universal parasitological confirmation prior to treatment with artemisinin-based combination therapy (ACT) became an internationally accepted malaria case-management standard in 2010 [1]. By the end of 2011, 41 of 44 malaria endemic countries in the WHO African Region had adopted the policy of parasitological diagnosis for all age groups in all epidemiological settings by either malaria microscopy or rapid diagnostic tests (RDTs) [2]. The “test and treat” policy for malaria has been recently extended to include a “track” component which should ensure that routine information systems capture and reliably report commodity stocks, testing of all suspected cases and subsequent appropriate treatment [3], [4]. Unfortunately, the adequacy and the quality of routine information systems across Africa is often characterized by complexities of revising systems to enable collection of desired indicators, suboptimal reporting rates and low data quality [5]–[8]. Therefore, across many African countries the monitoring information on the quality of malaria case-management is either absent or collected only periodically on limited scale by various research groups under rarely generalisable implementation scenarios [9].

In line with international recommendations, Kenyan MoH's Division of Malaria Control (DOMC) launched in 2009 universal “test and treat” case-management policy as a key component of the new 2009–2017 National Malaria Strategy (NMS) [10]. An accompanying Monitoring and Evaluation Plan recognized the importance of the malaria case-management quality, the weaknesses of routine information systems, and the importance of monitoring those indicators representing main deficiencies detected in the past which may compromise success of the new policy [11]. Prior to the policy change, studies from Kenya and other African countries suggested that ACTs and malaria diagnostics may be frequently out of stock [12]–[15]; where diagnostics are available febrile patients may not be tested [16]–[19]; patients with negative results may still receive antimalarial treatment [18]–[24]; and non-recommended therapies may be commonly used [25], [26] with suboptimal counseling and drug dispensing practices [26]–[29]. The persistence of such health systems and clinical practice deficiencies would compromise the cost-benefit of the “test and treat” policy and patients treatment outcomes [30]–[32].

Between 2010 and 2013, a series of regular, nationally representative surveys were undertaken to provide timely information to policy makers, implementers, and donor organizations on case-management progress. Here, we report key findings of the 2010 baseline survey undertaken prior to the beginning of the implementation activities under the new NMS and five follow up surveys of which the last one was undertaken in 2013, immediately prior to the mid-term performance review of the national 2009–2017 malaria strategy.

## Methods

### Kenyan malaria case-management policies and recommendations

**Figure 1:**
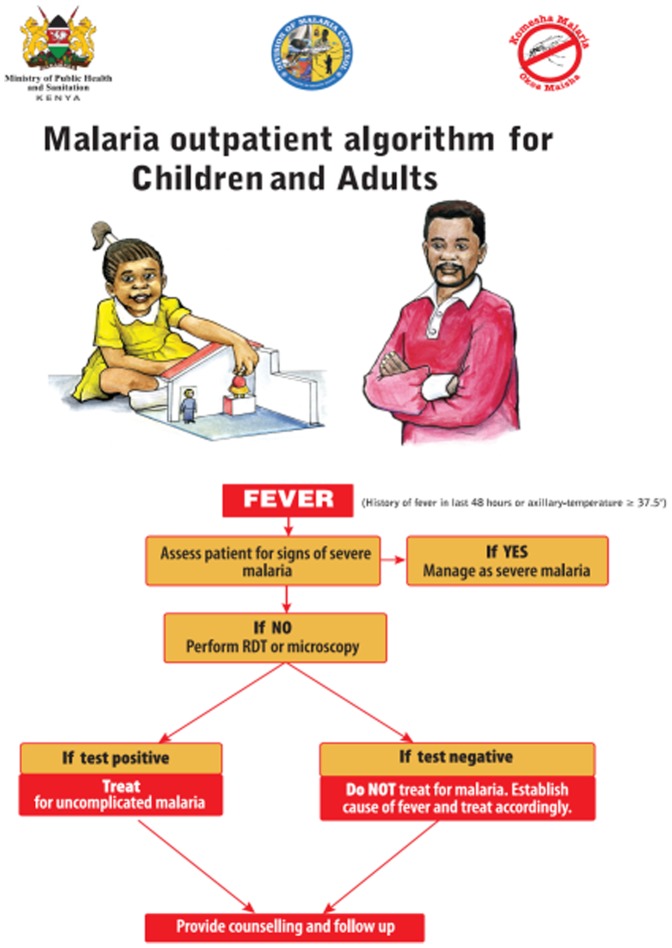
Kenyan “test and treat” policy translated into malaria outpatient algorithm.

In 2004, Kenya changed first-line treatment policy for uncomplicated malaria from sulfadoxine-pyrimethamine (SP) to a specific ACT, artemether-lumefantrine (AL) [33]. Quinine was recommended for special patient groups such as children below 5 kg, pregnant women, treatment failures, and for severe malaria [34]. In 2010, the treatment policy was further revised to recommend dihydroartemisinin-piperaquine (DHA-PPQ) for the second-line treatment, and the use of AL in the second and the third trimester of pregnancy and across all weight bands [35]. Finally, the 2012 policy revisions recommended parenteral artesunate for prereferral and severe malaria treatment while quinine remained recommended treatment only in the first trimester of the pregnancy [36]. Regarding malaria diagnosis, the policy before 2010 included age and endemicity specifics, which were abandoned in 2010 to the recommendation of universal parasitological testing of all febrile patients across all areas of varying endemicity with malaria microscopy or RDTs, and subsequent antimalarial treatment for only test positive patients [35] (Figure 1).

### Main 2010-2013 implementation activities

Between 2010 and 2013 the key activities relevant for the implementation of “test and treat” policy at public health facilities included procurement and distribution of antimalarial medicines and RDTs, development and distribution of new case-management guidelines and job-aids for health workers, three rounds of national in-service trainings for front-line health workers, and strengthening of supervisory and malaria microscopic capacities. The procurement and distribution of AL, the first time deployed to public facilities in 2006, continued between 2010 and 2013 through combined “push-pull” system depending on area and facility level before being entirely implemented in 2012 as “pull” system based on AL consumption. Drug management activities also included strengthening of routine logistics and management information systems through health workers training, routine supportive supervision, and on smaller scale, through implementation of innovative approaches to mitigate stock-outs at peripheral level [37]. By 2013, the DHA-PPQ second-line policy had not been implemented while procurement and distribution of parenteral artesunate had been undertaken only on a limited scale. The distribution of RDTs, initiated in 2006 in epidemic prone areas and subsequently in several other areas during various pilot projects [17], [37], continued erratically until the second half of 2012 when the first nationwide distribution took place.

The new national guidelines recommending “test and treat” policy were finalized in September 2010. They were supported by wall charts reflecting all age outpatient algorithms in February 2011, after which both job-aids were distributed to health workers through routine commodity supply channels and during in-service trainings. Three nationwide rounds of malaria case-management trainings for front-line health workers at public facilities have been undertaken. Firstly, between April and September 2010, 4,807 health workers were trained prior to the distribution of guidelines and job aids; secondly, 3,000 health workers were trained in November and December 2012, and finally, 1,950 between March and May 2013. All trainings followed training-of-trainers two-stage cascade format according to a standardized curriculum [38] in the workshop formats with teaching modalities including lectures, theoretical case scenarios and practicals on performing RDTs. Training duration was 3 days with one day devoted to the management of uncomplicated malaria; the exception being a one-day training in 2012 focusing only on uncomplicated malaria, RDTs and commodity management. Furthermore, malaria supervisory manuals emphasizing supportive supervision with observations of consultations were developed in 2010, piloted subsequently in one province and thereafter implemented on large scale either as stand-alone or through the integrated routine supervision [39]. Finally, quality of routine malaria microscopy was supported through in-service training of 140 microscopists and limited implementation of the quality assurance procedures mainly initiated through the non-governmental organizations.

### Study design, sample size and sampling

The study design included six cross-sectional health facility surveys in public facilities measuring levels and trends in national indicators on the coverage of health facilities and health workers with malaria related health systems support activities and the quality of outpatient malaria case-management in accordance with national guidelines. All surveys applied the same methodologies. The rational for indicators and the methodological details have been published previously [40]. Briefly, the key health systems support indicators included the proportion of health facilities with malaria diagnostic capacities (microscopy and/or RDTs), antimalarial medicines, national case-management guidelines and displayed wall charts. At the health worker level the key indicators were the proportion of health workers trained on “test and treat” case-management policy and receiving supervisory visit including malaria case-management activity during the three month period prior to the surveys. The primary study indicator was measured at the patient level and was defined as the composite case-management performance for febrile patients including all of the following testing and treatment criteria: 1) patient was tested for malaria; 2) if positive test result patient was treated with AL, and 3) if negative test result patient was not treated for malaria. The secondary indicators at patient level reflected individual components of the case-management including testing, treatment, dispensing and counseling practices in various patients' subgroups.

The sample size was calculated to provide national level estimates and detect 15% change in the performance of the primary case-management indicator between any of two survey points. The sample size was adjusted to take into consideration the effect of clustering at the health facility level and the likelihood of practices at facilities with unavailable case-management commodities. In order to detect 15% difference (from conservative estimates of 50% to 65%) with the confidence level of 5%, power of 80%, a design effect of 2, and an assumption that 50% of facilities will not have either AL or malaria diagnostics, the estimated sample size was 680 patients below and above 5 years of age during each survey. Estimating a minimum average of 4 eligible patients in each age group at each facility over one survey day, the minimum required number of surveyed facilities was 170 (680/4). The indicators at the facility level were not subjected to the cluster effect and the sample of 170 facilities was estimated to be sufficient to detect minimum difference of 15% (from 50% to 65%) with the same power (80%) and level of confidence (5%) as for the measurements of indicators at the patient level.

The national representativeness was assured drawing a stratified random sample taking into consideration administrative boundaries, type of facilities and their ownership. From the universe of 6,094 public facilities the following categories of facilities were excluded from the sampling frame: 1) facilities from Nairobi province due to absence of malaria transmission and requiring special studies to evaluate malaria case management, 2) tertiary hospitals since they serve mainly as referral facilities, and 3) facilities run by other than Ministry of Health (MoH) and Local Authorities (LA) because they provide services to special patient groups such as military or prisoners. In total, 861 facilities were excluded and therefore our sampling frame consisted of 5,233 health facilities. For the purpose of sampling, facilities belonging to the faith based and non-governmental organizations were classified into one category. Similarly, MOH and LA owned facilities were grouped into the government category as well as smaller facilities such as dispensaries and health centres which also represented one category. Therefore, in each of seven Kenyan provinces, four strata based on the facility type (hospitals versus smaller facilities) and ownership (government versus faith based/non-government) were formed. Finally, from each of the 28 formed strata, a simple, random sample of facilities proportional to the contribution of the stratum size (sampling fraction) to the universe of facility was drawn. Prior to each survey the most updated list of facilities was used and the same sampling strategy was applied with the exception of the last 3 survey rounds when additional 143 facilities from North Eastern province were excluded from the sampling frame due to insecurity in areas afflicted by the war conflict in the neighboring Somalia.

### Data collection

Following five days of the training which was conducted prior to each survey, the field data collection was undertaken over four weeks with ten teams composed of three surveyors. Data were collected over one survey day at each facility. All outpatients presenting during daytime operating hours underwent rapid screening when they were ready to leave the facility. Non-referred and non-pregnant patients presenting for an initial visit with fever and weighing ≥5 kg proceeded with an interview. During the interviews information was collected about patients' age, weight, sex, temperature, duration of fever, main complaints, prior use of antimalarial drugs, and the basic assessment, drug dispensing and counseling tasks performed during the facility visit. From patient-held cards information was also collected about malaria diagnostics requested, results reported and medications prescribed. In Kenyan context the patient cards are the mandatory outpatient documents and present the main communication link between clinicians, laboratories and pharmacy. During the survey day each facility was assessed to determine the availability of antimalarial drugs, RDTs, and functional malaria microscopy service on survey day and retrospectively for the period three months prior to the surveys. The availability of weighing scales, case-management guidelines and displayed wall charts was also established. At the end of the working day all health workers who saw recruited patients on the survey day were interviewed to collect information on their demographics, pre-service training, access to guidelines, and exposure to in-service training and supervision. All data collection tools are available upon request to the authors.

### Analytic approaches and statistics

Descriptive analysis measuring levels and trends was performed at the health facility, health worker, and patient level. First, to assess the health systems readiness to implement new case-management policy the analysis was undertaken at the health facility and health worker level. Second, to assess national performance of the 2010 “test and treat” policy, health workers' practices were analyzed at the patient level at all survey facilities regardless of the availability of AL and malaria diagnostics. Third, to assess health workers adherence to the guidelines and recognizing that absence of commodities preclude case-management practices, the same patient level analysis was restricted to health facilities with available AL and malaria diagnostics. Fourth, to assess the quality of AL dosage prescriptions, and the quality of dispensing and counseling practices, the analysis was respectively restricted to patients who had AL prescribed and to those who had both, AL prescribed and dispensed at the facility. Fifth, to examine health workers adherence to the “test and treat” policy in relation to the type of malaria diagnostics, the stratified analyses by the respective availability and use of RDTs and malaria microscopy were performed during the last survey. Sixth, to assess the impact of the new policy on the consumption of medicines, trend analysis on overall use of AL and antibiotics was performed. Finally, the results on the key case-management indicators were stratified for patients below and above 5 years of age.

Data entry was undertaken in Microsoft Access and all questionnaires were entered twice with data files compared for errors using a verification programme and referring to original paper-based questionnaires. All analyses were performed using STATA 11 (StataCorp, College Station, Texas). Level estimates are calculated for each survey as proportions with corresponding 95% confidence intervals (CI). Chi-square test for comparison of proportions was used to compare baseline results with results of the last follow up survey. Chi-square test for linear trend was used to assess significance in trends across all survey rounds. Health facility level analyses were not subjected to clustering however at the patient level, 95% CIs for level estimates and p-values for Chi-square test for comparison of proportions were adjusted for clustering at the health facility level while significance in trends across six survey rounds was adjusted for facility level clustering using mixed effect logistic regression with survey round being independent variable in the model. Hypothesis testing and CI estimations were done with an alpha level of 0.05.

### Ethics statement

Ethical approval for the study was provided by the Kenyatta National Hospital/University of Nairobi-Ethics and Research Committee (KNH-ERC/A/383). Informed written consent was obtained for all participating patients and health workers.

## Results

### Description of samples

Table 1 shows survey dates and study samples for the main study populations at each survey round. In summary, the number of assessed facilities across six surveys ranged between 172 and 176, interviewed health workers between 216 and 237, evaluated outpatient consultations at all facilities between 1,208 and 2,408, and those at facilities with available commodities between 634 and 1,302. Of 1,357 health workers interviewed during all survey rounds, only 30 (2.2%) have been repeatedly interviewed and none of them more than twice. The characteristics of facilities, health workers and patients were similar across six survey rounds. During all surveys, the majority of facilities were dispensaries (range: 63.8%–70.1%), followed by health centres (range: 18.4%–25.0%) and hospitals (range: 10.5%–12.2%). Regarding ownership, the majority were government owned (range: 73.0%–78.4%) followed by faith-based (range: 19.3%–25.9%) and non-governmental (0.6%–2.3%) facilities. The average health workers' age ranged across surveys from 34 to 37 years, the majority were female (range: 52.7%–59.9%) and nurses by profession (range: 58.8%–65.6%). The second most common category of health workers' cadres were clinical officers (range: 28.3%–31.4%) while doctors were very rare (range: 0.9%–1.7%). With respect to febrile patients, most were female (range: 53.8–58.1%), 5 years and older (range: 53.6%–59.0%), and without prior use of any antimalarial during the current illness (range: 95.0%–96.7%). Less than half of the patients had axillary temperature ≥37.5°C (range: 23.8%–35.1%) on survey day with negligible proportion completing AL dose prior to the health facility visit (range: 0.5%–1.2%).

**Table 1:** Number of health facilities assessed, health workers interviewed and outpatient consultations evaluated for febrile patients at all facilities and facilities with commodities in stock, by survey.

Survey	HF	HW	OPD consultations at all HFs		OPD consultations at HFs with Dx and AL in stock	
			<5 years	≥5 years	<5 years	≥5 years
Baseline (Jan-Feb 2010)	174	224	1,070	1,335	591	648
Follow-up 1 (Nov-Dec 2010)	176	237	675	781	420	441
Follow-up 2 (July-Aug 2011)	174	233	535	673	301	333
Follow-up 3 (Mar-Apr 2012)	172	220	581	710	340	428
Follow-up 4 (November 2012)	172	216	510	735	383	536
Follow-up 5 (June 2013)	172	227	592	839	549	753

HF = health facility; HW = health worker; OPD = outpatient department; Dx = diagnostics; AL = artemether-lumefantrine.

### Health facility and health worker readiness to implement “test and treat” policy

**Figure 2:**
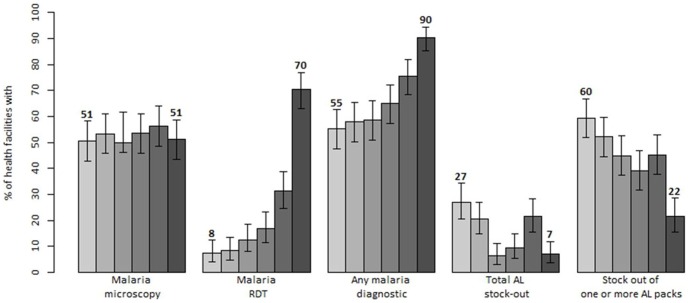
Trends in key health facility indicators reflecting availability of malaria diagnostics and ACTs in Kenya: results of six national surveys between 2010 and 2013 (each bar corresponds to a different survey).

**Figure 3:**
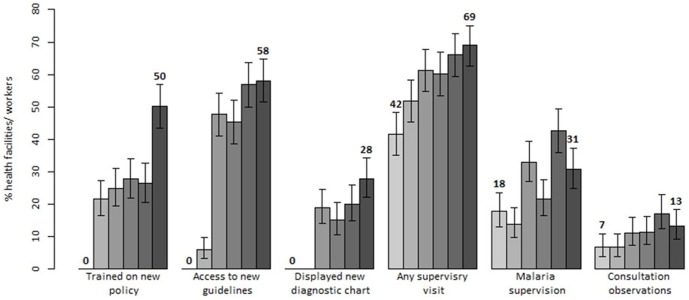
Trends in indicators reflecting coverage of health facility and health workers with key health systems support activities in Kenya: results of six national surveys between 2010 and 2013 (each bar corresponds to a different survey).

Figures 2 and 3 present levels and trends of the key health systems support indicators reflecting health facility and health workers readiness to implement “test and treat” case-management policy. The baseline results showed that prior to the policy implementation the major contributor to the parasitological diagnosis of malaria was microscopy, available at 50.6% (95% CI: 43.1–58.1) of facilities. There were no significant changes in the provision of malaria microscopy over the monitoring period (p = 0.719). Yet, a significant increase in the overall parasitological capacities was observed between the baseline and the last follow-up survey mainly due to the major 62.3% increase in the availability of RDTs reaching a coverage of 69.8% (95% CI: 62.8–76.7) by the time of the last survey. At facilities with microscopy, there was modest 9.3% increase in the facilities receiving quality control visit while at facilities with RDTs there was 14.7% increase in the supervisory visits on the use of RDTs. However, at these facilities coverage with quality control activities at the end of the monitoring period was still low for both diagnostic services: 18.4% (95% CI: 11.5–28.2) for microscopy and 20.0% (95% CI: 13.7–28.2) for RDTs.

Stock assessments of medicines on survey days showed that availability of at least one AL pack was relatively high (range: 89.1%–97.2%) however facilities less commonly had all four packs in stock (range: 45.4–71.5%). Importantly, significant declining trends were observed in all AL stock-out indicators when stock-out was measured as retrospective absence of the commodity in the duration of 7 or more consecutive days over a 3 months period prior to the surveys (Figure 2). Comparing the baseline results with results of the last follow up survey, total AL stock-out decreased from 27.2% to 7.0% (p<0.001) while stock out of one or more AL packs declined from 59.5% to 21.6% (p<0.001). Stock-outs of individual AL packs ranging from 37.6% to 52.0% prior to the baseline decreased to between 14.6% and 21.6%. The declining trends across six survey rounds were statistically significant for all AL stock-out indicators (p<0.001). With respect to other antimalarials, DHA-PPQ and injectable artesunate, which were not available at baseline, were respectively stocked at only 4.1% and 20.3% of facilities during the last survey. Conversely, facilities were equally stocked with quinine tablets (80.8%) and quinine injections (80.2%) without significant changes over the monitoring period.

During the baseline survey, there were neither trained health workers on the new case-management policy nor health facilities providing access to new guidelines and “test and treat” diagnostic wall charts (Figure 3). At the time of the last survey, coverage of trained health workers had reached 50.2% (95% CI: 43.7–56.8) with 58.1% (95% CI: 50.7–65.6) of facilities having new guidelines and 27.9% (95% CI: 21.1–34.7) displaying wall charts. The trends in the training, guidelines and wall charts coverage reflected implementation timing of these activities. Yet, despite a declining trend, the findings during the last survey revealed that 25.6% of facilities displayed obsolete algorithms promoting presumptive treatment in children while 56.7% had old malaria guidelines providing the same recommendations. During the last survey, 30.2% of facilities were found with both, valid and obsolete, guidelines. Finally, coverage of supervised health workers increased from 41.5% prior to the baseline to 69.2% prior to the last survey (p<0.001). There was also a modest increase in the coverage of health workers with supervisory visits including malaria case-management and with the visits including consultation observations. The end-line coverage with these indicators was however still low reaching respectively only 30.8% and 13.2% of health workers (Figure 3).

### Composite case-management performance and testing rates

**Figure 4:**
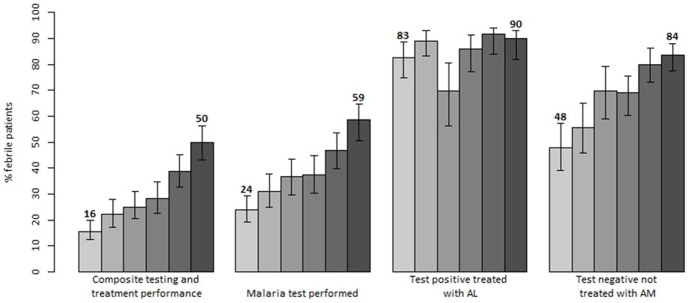
Trends in key diagnostic and treatment indicators reflecting performance of the new case-management policy in Kenya: results of six national surveys between 2010 and 2013 (each bar corresponds to a different survey).

The composite performance, defined as patient tested for malaria and treated with AL if the test result was positive or not treated for malaria if the result was negative, improved significantly by 34.2%; from 15.7% at the baseline to 49.9% at the last follow-up survey (p<0.001) with an increasing trend across all survey rounds (p<0.001) (Figure 4). A similar trend was observed in children below 5 years (11.8% vs 49.0%; p<0.001) and in patients 5 years and above (18.9% vs 50.5%; p<0.001). With respect to testing rates, improvements of 34.0% were observed; from 23.9% at the baseline to 57.9% during the last survey (p<0.001). In the same period, the testing rates in children increased from 20.5% to 55.2% (p<0.001) while testing in older children and adults improved from 26.7% to 59.7% (p<0.001).

In the subset of facilities where AL and malaria diagnostics were available, the baseline values were higher, improvements smaller but reaching higher end-line performance values. At these facilities, the composite performance improved by 26.4%, from 28.1% at the baseline to 54.5% (p<0.001) during the last survey, while testing improved by 20.7%, from 42.5% to 63.2% (p<0.001). With respect to age, the composite performance in children improved from 19.3% to 52.5% (p<0.001) while testing increased from 33.3% to 59.0% (p<0.001). In patients 5 years and older improvements were lower compared to young children, however the composite performance still significantly improved from 36.1% to 56.0% (p<0.001) while testing rates increased from 50.8% to 66.3% (p = 0.006).

Results of the last follow-up survey stratified by exclusive availability of RDTs and malaria microscopy revealed no significant differences in the composite performance (46.5% vs 51.1%; p = 0.578) and testing rates (54.5% vs 57.5%; p = 0.728); however health workers adherence was significantly higher at facilities providing both diagnostic services. At these facilities the composite performance was 65.6% while 76.3% of febrile patients were tested. Notably, at facilities providing both diagnostic services, significantly higher proportion of tested patients had malaria microscopy performed (67.3%) compared to RDTs (30.8%) while only 1.9% of patients had both tests performed.

### Treatment practices by use and result of malaria test

Stratified treatment analysis by the use and result of malaria test at facilities with available commodities revealed several improvement patterns over the monitoring period. First, despite relatively high baseline adherence, an improvement was observed in the use of recommended AL treatment for test positive patients (83.3% vs 90.3%; p = 0.138). During the last survey, 95.2% of children below 5 years were prescribed AL while non-recommended treatments comprising either quinine monotherapy or combination of AL and quinine, became uncommon and mainly prescribed for older children and adults where lower adherence for test positive patients was observed (87.4%; p = 0.074). There was no difference in AL treatment between patients tested positive with RDTs or malaria microscopy (90.9% vs 89.1%; p = 0.727). Second, among patients with negative test result, a substantial declining trend in the proportion of patients treated for malaria was observed. Health workers' treatment adherence to negative test results improved by 36.2% and by the time of the last survey there were relatively low levels of non-adherence to test negative results (16.6%) compared to the baseline results (52.1%; p<0.001). A significant decline in this non-adherent practice was observed in both age groups; 41.6% (56.7% vs 15.1%; p<0.001) in children below 5 years and 31.1% in older children and adults (48.7% vs 17.6%; p<0.001). There was no difference in non-adherent practices for test negative patients tested with either RDTs or malaria microscopy (17.5% vs 15.8%; p = 0.745). Finally, a significant decline of 44.5% of antimalarial prescriptions was observed among patients not tested for malaria. This has resulted in 19.2% of these patients treated for malaria during the last survey, nearly all with AL therapy.

### Quality of AL dosing, dispensing and counseling

**Figure 5:**
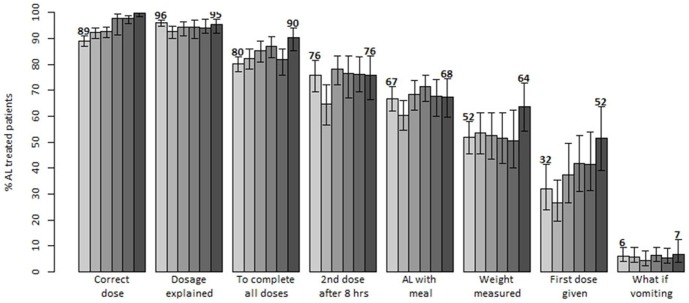
Trends in the quality of health workers AL dosing, dispensing and counseling practices in Kenya: results of six national surveys between 2010 and 2013 (each bar corresponds to a different survey).

The quality of AL dosing, dispensing and counseling was evaluated for eight performance tasks (Figure 5). The baseline values for correct dosing were high (89.2%). Yet, a significant improvement trend in recommended dosing was observed. During the last follow-up survey nearly all patients (99.8%) were correctly dosed for their weight. With respect to the seven dispensing and counseling tasks, the comparison between baseline and the last survey showed significant improvements for the following three tasks: weighing of patients (51.8% vs 64.1%; p = 0.039), administration of the first AL dose at the facility (32.1% vs 51.5%; p = 0.013) and provision of advice that all doses should be completed (80.3% vs 90.4%; p = 0.002). Weighing was more common for children than adults and at the time of the last survey three-quarters (75.7%) of children were weighed. However, no improvements were observed in the performance of the remaining four tasks (Figure 5). Of these tasks, three (advice on correct dosing, advice on the second dose after 8 hours and advice on taking AL after a meal) were performed for more than two-thirds of the patients while the only counseling task that was rarely performed throughout the monitoring period was provision of advice on what to do in case of vomiting (Figure 5).

### Overall use of AL and antibiotics

The consumption of AL for febrile patients decreased by 27.9% over the monitoring period, from 63.5% at the baseline to 35.6% at the last follow up survey (p<0.001) with significant declining trend over all survey rounds (p<0.001). A significant decline in AL use was observed in children below 5 years of age (65.4% vs 31.6%; p<0.001) and in patients 5 years and older (62.1% vs 38.5%; p<0.001). Throughout the monitoring period the overall use of antibiotics was very high (survey range: 75.4%–77.6%) and without significant changes between the baseline and the last survey round (75.4% vs 77.4%; p = 0.331).

## Discussion

We have reported results of six rounds of national health facility surveys undertaken between 2010 and 2013 at public facilities in Kenya. The surveys measured health systems readiness and quality of malaria case-management prior to and following implementation of the “test and treat” policy in Kenya. The findings revealed major improvements in key indicators during this period but also gaps towards the optimistic universal targets set by the 2009–2017 National Malaria Strategy.

### Health facility and health worker readiness to implement “test and treat” policy

The basic determinant of effective malaria case-management is the availability of “test and treat” commodities. By mid 2013, the capacity of Kenyan facilities to provide parasitological diagnosis reached 91%, a direct result of the national RDT roll-out whose distribution started in 2012. The sustained coverage will be however critically dependent on the maintenance of the effective, rather complex RDT supply chain [41]. Experiences from other countries [42] and from 2008 in Kenya with AL, have shown that supply chain interruptions can result in massive stock-outs [13] compromising policy implementations and patient outcomes [43]. Despite an international focus on RDTs, malaria microscopy as the traditional diagnostic mainstay in Kenya deserves attention. We found approximately half of the facilities providing malaria microscopy; the results mirroring the recent surveys in Kenya [42] and being higher than in many other African countries [42]. The use of microscopy is also high, and indeed at facilities with both diagnostic services, it was preferentially used compared to RDTs. There has been no recent study evaluating the quality of microscopy in Kenya but over-reporting of test positive results reported earlier [21], low levels of quality control visits found at the surveyed facilities and substantial availability and microscopy use in the country call for urgent implementation of the quality assurance programmes in line with existing national laboratory manuals. Yet, some results of this study showing usual “gold standard” pattern of slightly lower test positivity rates of microscopy compared (37%) to RDTs (45%) might indicate that the quality of microscopy could be better than previously reported. However, confirmations of these findings require studies including gold standard examinations for both RDTs and routine microscopy.

Not unique to Kenya, AL stock-outs with detrimental consequences have been frequently reported in the past [43]. We found substantial declines in all AL stock-out indicators resulting in relatively low percentage (7%) of facilities unable to provide first-line therapy. Several factors may have contributed to these improvements. First, the transition during this period to the country wide “pull” system based on AL consumption and ordering through the integrated electronic logistic information systems may have improved the supply chain. Secondly, some strengthening of the drug management practices at peripheral facilities through health workers training and district capacities to respond to threatening stock-outs via redistribution of commodities also took place in this period [37]. Finally, and probably most importantly, the results of this study have clearly shown that following the implementation of “test and treat” policy in Kenya, the overall AL use for febrile patients has massively declined from 64% at the baseline to 36% in 2013. This inevitably extended peripheral AL stocks even under the scenarios of imperfect supplies. Similar reductions in AL use have been reported from Senegal [44] and Zambia [45]; the countries reporting successful implementation of “test and treat” policy. There is, however, a need for large scale procurement and distribution of recommended therapies for treatment failures (DHA-PPQ) and severe malaria (parenteral artesunate). Despite the policy shifts in 2010 to DHA-PPQ and in 2012 to artesunate, and provided training on the drug use to health workers, we found very low availability of these commodities, a similar position to the lengthy policy-to-practice translation processes following first-line drug changes reported in Kenya [26] and in other African countries [15], [46].

By mid 2013, we found half of health workers had been trained on the “test and treat” policy - the coverage reflecting programmatic reports of 9,757 trained health workers between 2010 and 2013 among an estimated national universe of 20,000 front-line health workers. An important observation was the increasing trends in supervision; however despite two-thirds of health workers receiving supervision less than one-third had a visit that included any malaria case-management activity while observations of consultations as a qualitative marker of the supervision was rarely performed. The observed pattern might be due to the integration of supervisory activities across diseases, promoted during the monitoring period, and which may have contributed to an increased overall coverage but might have resulted in neglecting components on malaria case-management.

### Malaria case-management

Over three years of the monitoring period, we found significant improvements in all case-management indicators reflecting health workers adherence to “test and treat” policy. First, testing rates increased by 34% reaching 58% of tested febrile patients at all facilities and 63% tested at facilities with available diagnostics. The testing levels were higher compared to reports from Zambia [18] and Angola [47], similar to those from several Tanzanian studies [9], [48]–[50] but lower compared to what can be achieved under more controlled, smaller scale conditions in Uganda [51]. Notably, at facilities providing both diagnostic services we found significantly higher testing rates (76%), preference for malaria microscopy, and in contrast with our previous findings [40], there was no difference in testing rates between facilities providing solely RDTs (55%) or malaria microscopy (58%). The findings suggest that (1) health workers have overcome an initial reluctance to use RDTs, (2) when diagnostic options exist, malaria microscopy is perceived as a gold standard method, and (3) deployment of RDTs to facilities with microscopy is the appropriate strategy that can boost testing rates. The latter could be of particular importance to address the workload limitations, an important diagnostic performance factor as shown in our earlier analyses and in other studies [16], [40], [47].

With respect to treatment practices in relation to test result, we found that treatment for test positive patients became increasingly the norm, with over 90% of patients treated with AL. This adherence is significantly higher than found in Kenya during early AL implementation phase [26], in accordance with several reports in other countries [24], [52]–[54] however at variance with recent reports from Tanzania [9], [49] and Sudan [46] where drug rationing due to stock-outs and preferences for injectable therapies may have contributed to higher non-adherent practices. We also found that antimalarial treatment for test-negative patients, one of the main compromising factors of “test and treat” policy reported across Africa [22]–[24], has declined by 34% reaching imperfect but more tolerable rates of 16%. During the past 10 years we have observed a steady decline in this irrational practice in Kenya – from as high as 79% found in 2003 [21], over 67% in 2006 [19] and 53% in 2010 [40] to the current levels of 16% in 2013. The latest findings show no difference in adherence levels with respect to age and type of malaria test performed. The results are encouraging and suggest that the effects of the long-term policy promoting presumptive treatment of young children have largely waned over time [40] and that health workers' lack of trust in negative RDT results is less of a problem than previously observed.

Less success was however observed in correct AL dispensing and counseling practices - the key components of case-management determining patients' adherence to medication [55]–[57] and treatment outcomes [58]. While correct dosing for weight had become a norm, two underperformed dispensing and counseling tasks deserve attention. First, despite having AL dispensed, nearly half of the patients and equally children and adults, leave the facility without being given the first dose at the facility. This practice does not only delay prompt treatment but also misses opportunities to demonstrate administration of dispersible AL to children and therefore increases the risk of inappropriate administration at home [56]. Second, febrile malaria patients, and in particular children, often vomit after taking medicines, however they are rarely advised that if vomiting takes place within 30 minutes that they should take another dose and return to the facility for the replacement dose to complete the therapy. The possible reasons for these practices include, among others, lack of potable water at the facility, accountability for replacement medicines and possible conflicting messages between prompt treatment and administration after a meal. Future qualitative research is required to better understand these practices and their effects on patients' adherence.

### Consumption of AL and antibiotics

Finally, due to increased parasitological capacities and more rational prescribing of antimalarials, overall use of AL nearly halved between 2010 and 2013. Following RDT roll-out the decline in AL consumption has been well documented in several studies across Africa [9], [44], [45]. However in contrast with other studies [9] we have not observed a compensatory increase in the use of antibiotics, mainly due to already high use of antibiotics for febrile patients prior to the policy change. We have not evaluated appropriateness of antibiotic use, however given the findings of other studies on the presence of criteria for antibiotic use [59] there is a high likelihood of significant over-treatment. The need for better diagnostics for non-malarial fevers, development of guidelines for management of non-malaria febrile illness and their incorporation in malaria case-management trainings for health workers should be an urgent priority to rationalize not only antimalarial use but also the use of antibiotics.

### Programmatic implications and conclusions

The Kenyan DOMC is the only African malaria control programme successfully undertaking relatively simple, regular, facility-based malaria case-management surveys to track implementation of “test and treat” policy and circumvent deficiencies of the routine logistics and health information systems to provide desired and valid indicators. Importantly, throughout the process between 2010 and 2013, the findings of the surveys were not only disseminated to national and sub-national managers but also to large numbers of health workers during the series of national in-service trainings and supervisory activities. This may have also contributed to the improvements in malaria case-management observed over this period despite the activity being primarily initiated as the monitoring tool and not a quality improvement intervention. We are glad to reveal that by mid 2013 nearly all key indicators around “test and treat” policy for malaria have shown significant improvements. Yet at the time of the mid-term policy performance review, some important gaps towards universal targets still remain. Only the quantity and the quality of health systems interventions in the next 2014–2017 period accompanied with continued and close monitoring as demonstrated here will minimize some of the existing gaps. Kenya is currently undergoing the process of devolution where most of malaria control activities will be decentralized to 47 newly formed counties. In this new context, the national surveys measuring policy progress will remain the responsibility of national bodies; however, there is also need to explore modalities and feasibility of implementing county level surveys, perhaps as the quality improvement intervention starting with pilot counties expressing interests, with available funding, and having in place human capacities requiring minimal external technical input. Other countries in Africa facing similar needs and challenges may also consider health facility surveys as a tool to monitor malaria case-management.
